# Ewing’s Sarcoma of the Zygoma: A Very Rare Location

**DOI:** 10.7759/cureus.35793

**Published:** 2023-03-05

**Authors:** Joana Gonçalves, Hugo Marques, Rute Saleiro, Ângela Ferreira, Andreia Ferreira

**Affiliations:** 1 Maxillofacial Surgery Department, Centro Hospitalar e Universitario de Santo Antonio, Porto, PRT

**Keywords:** tumor, neoplasm, ewing´s sarcoma, facial bones, zygoma, sarcoma

## Abstract

Ewing’s sarcoma is a rare and aggressive neoplasm that typically affects the long bones. The presence of a primary tumor in the facial bones is extremely uncommon. Here, we present a case of a 21-year-old male with Ewing’s sarcoma of the zygoma. To date, only a few such cases have been reported worldwide in the literature.

## Introduction

Ewing’s sarcoma is a highly malignant neoplasm that typically affects the long bones [1,2]. The presence of a tumor in the facial bones occurs in only 1-4% of cases [3]. Zygoma infections are even rarer, with only a few cases reported in the literature. They are most common among young adult males [1]. Here, we present a case of Ewing’s sarcoma of the zygoma that was treated with chemotherapy, radical surgery, and primary reconstruction.

## Case presentation

**Figure 1:**
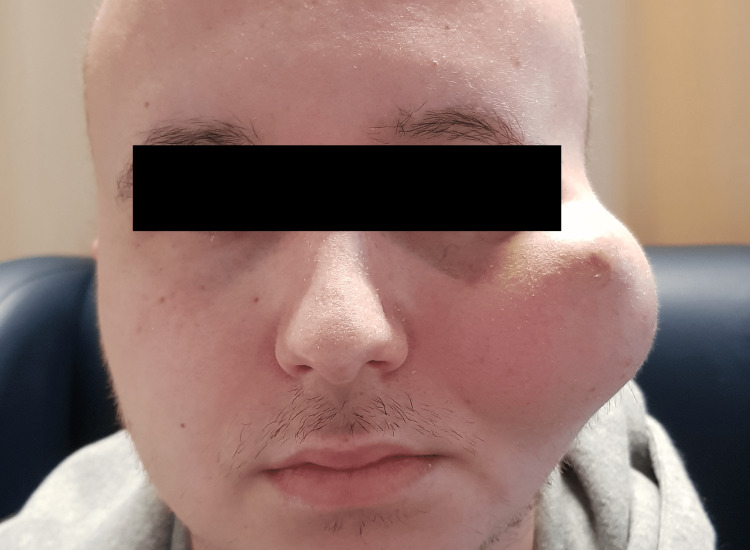
An enlarging mass in the left malar region

A 21-year-old male presented with an enlarged mass in the left malar region after seven months of evolution (Figure 1). The medical history was not noteworthy.

Computed tomography revealed a large lesion (6.5x5x4 cm) with an obvious origin in the zygomatic arch region. The lesion invaded the masseter muscle by projecting into the subcutaneous cellular tissues of the left maxillary region and the masticator space between the ramus of the mandible and the zygomatic arch. No ganglion formations was suspected to be malignant.

The magnetic resonance imaging (MRI) revealed a voluminous lesion on the left side, centered on the malar region and zygomatic bone, with lobulated contours, a heterogeneous signal on T1 and T2, and heterogeneous contrast product uptake. An incisional biopsy was performed. Microscopically, the connective tissue fragments were infiltrated with a malignant neoplasm composed of small monomorphic cells. Mitosis and necrosis were also observed. The immunohistochemical findings included anti-cytokeratin (CAM 5.2)-, special AT-rich sequence-binding protein 2 (SATB2)-, NKX2.2+, and CD99+ cells. *EWSR1 *gene rearrangements were detected using fluorescence in situ hybridization (FISH) (GEN21-0382). The patient was diagnosed with Ewing's sarcoma. There was no evidence of metastasis.

**Figure 2:**
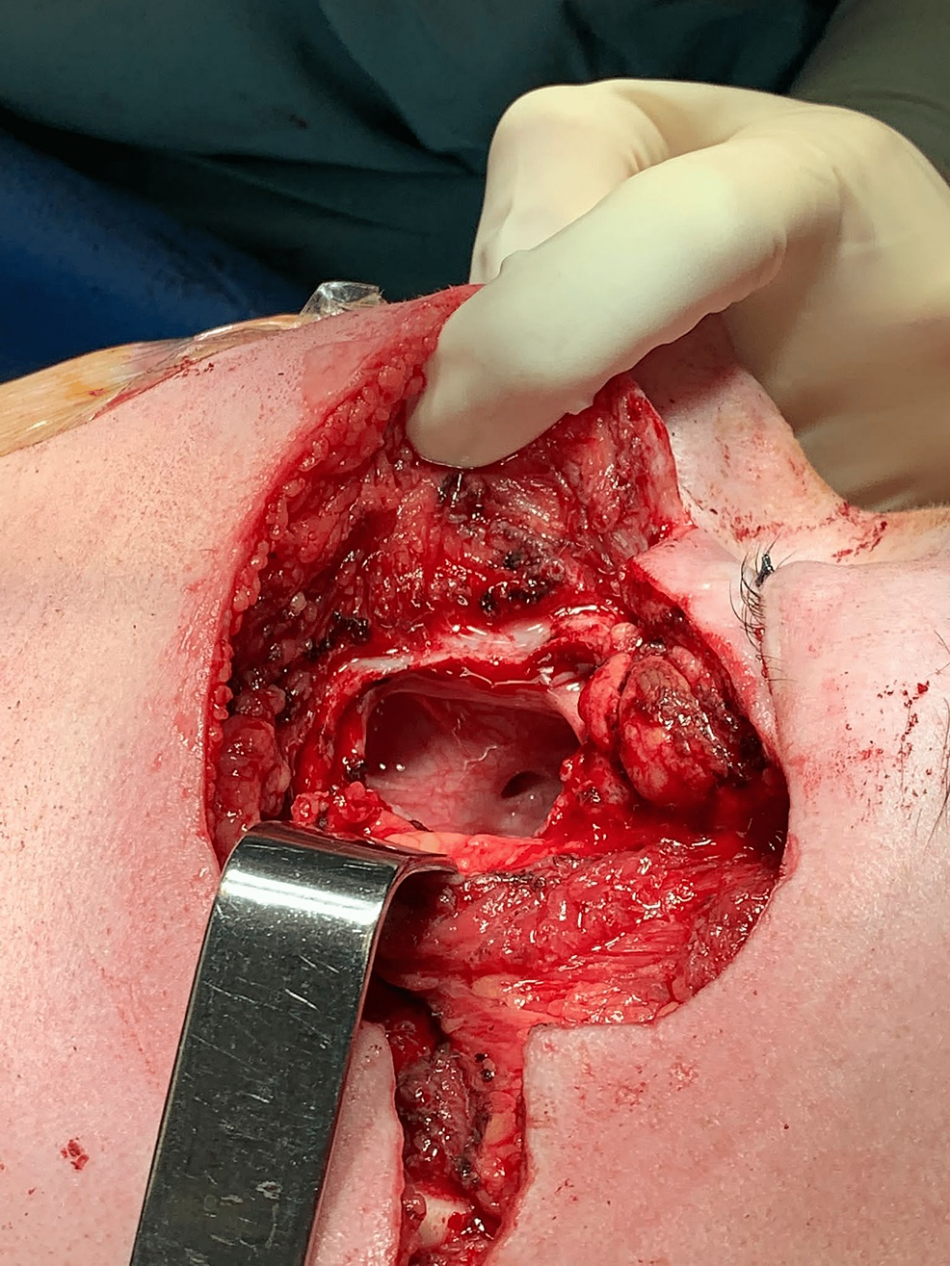
Image showing a defect after tumor resection

The patient received four cycles of neoadjuvant chemotherapy with vincristine, doxorubicin, and cyclophosphamide (VDC). On the other hand, clinical progression of the lesion was reported. Bloc tumor resection was performed with a section of the zygoma in the frontal and temporal buttress, orbital floor, and maxilla. Moreover, the V and VII nerves are also sacrificed (Figure 2).

**Figure 3:**
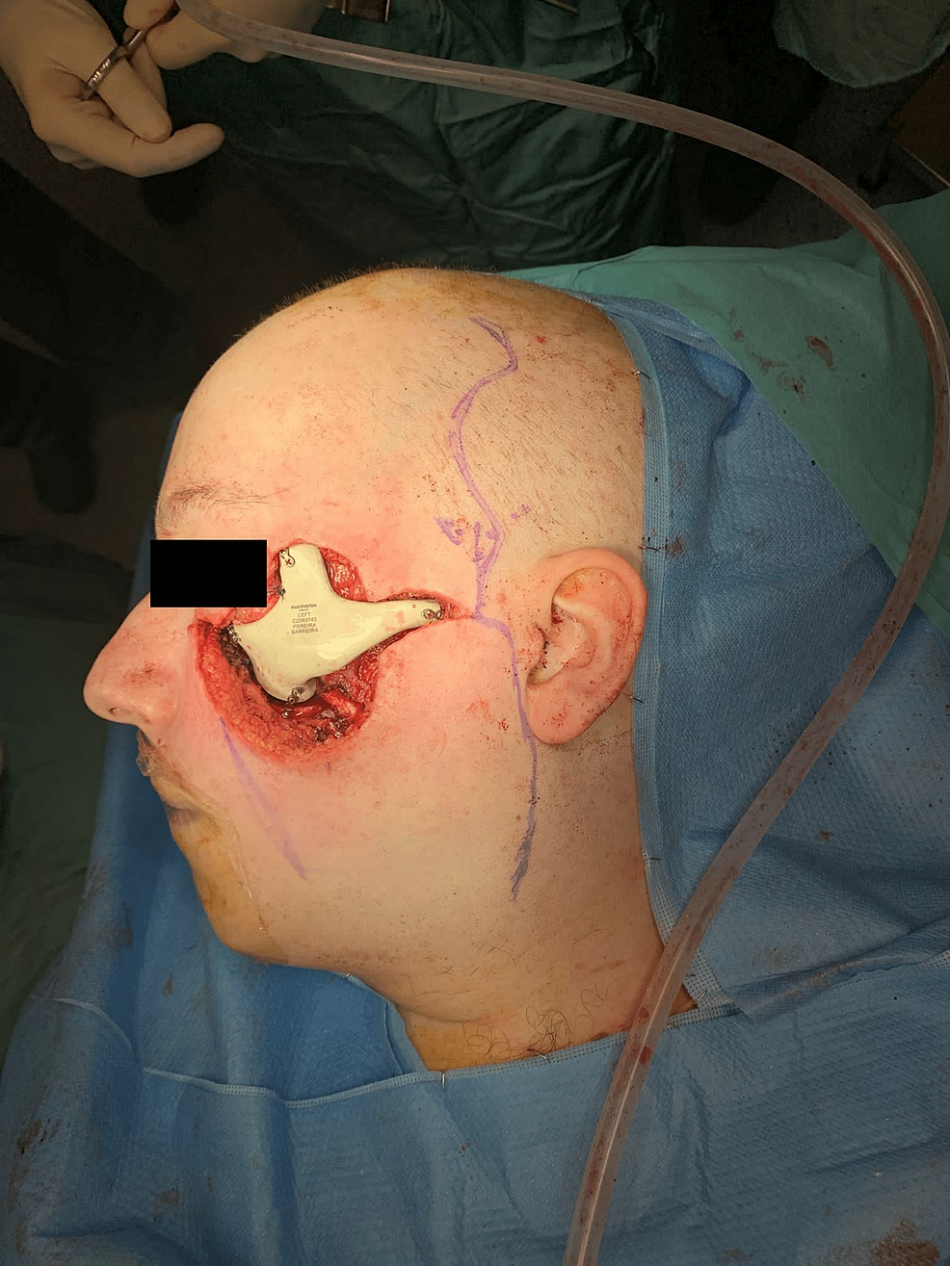
Defect reconstruction with a polyetheretherketone (PEEK) prosthesis

The facial defect was repaired using a polyetheretherketone (PEEK) prosthesis attached to a four-point fixation with plates and screws (Figure 3). 

**Figure 4:**
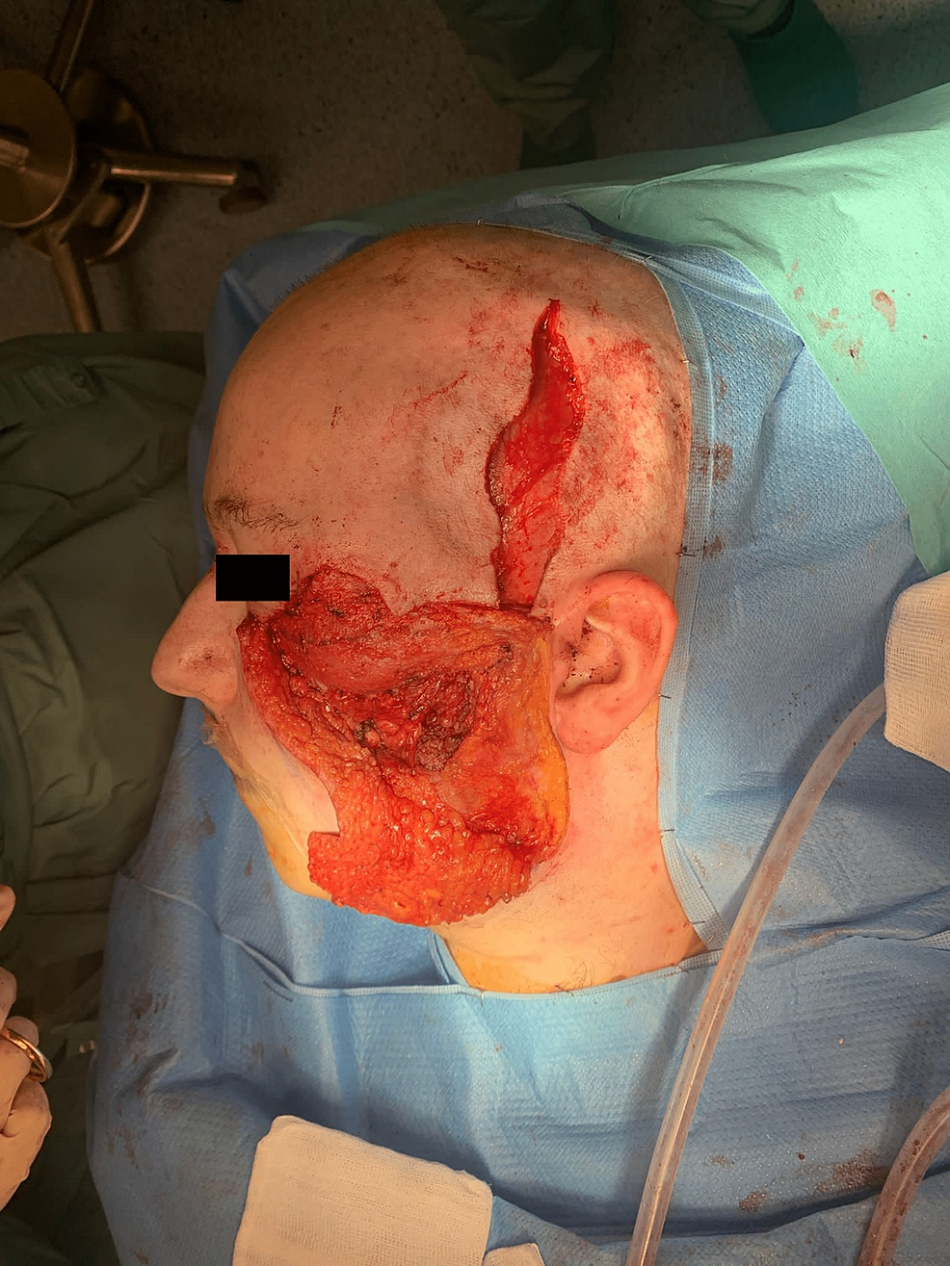
Temporal fascia flap covering the malar prosthesis

The malar prosthesis was wrapped in a temporal fascial flap (Figure 4). 

**Figure 5:**
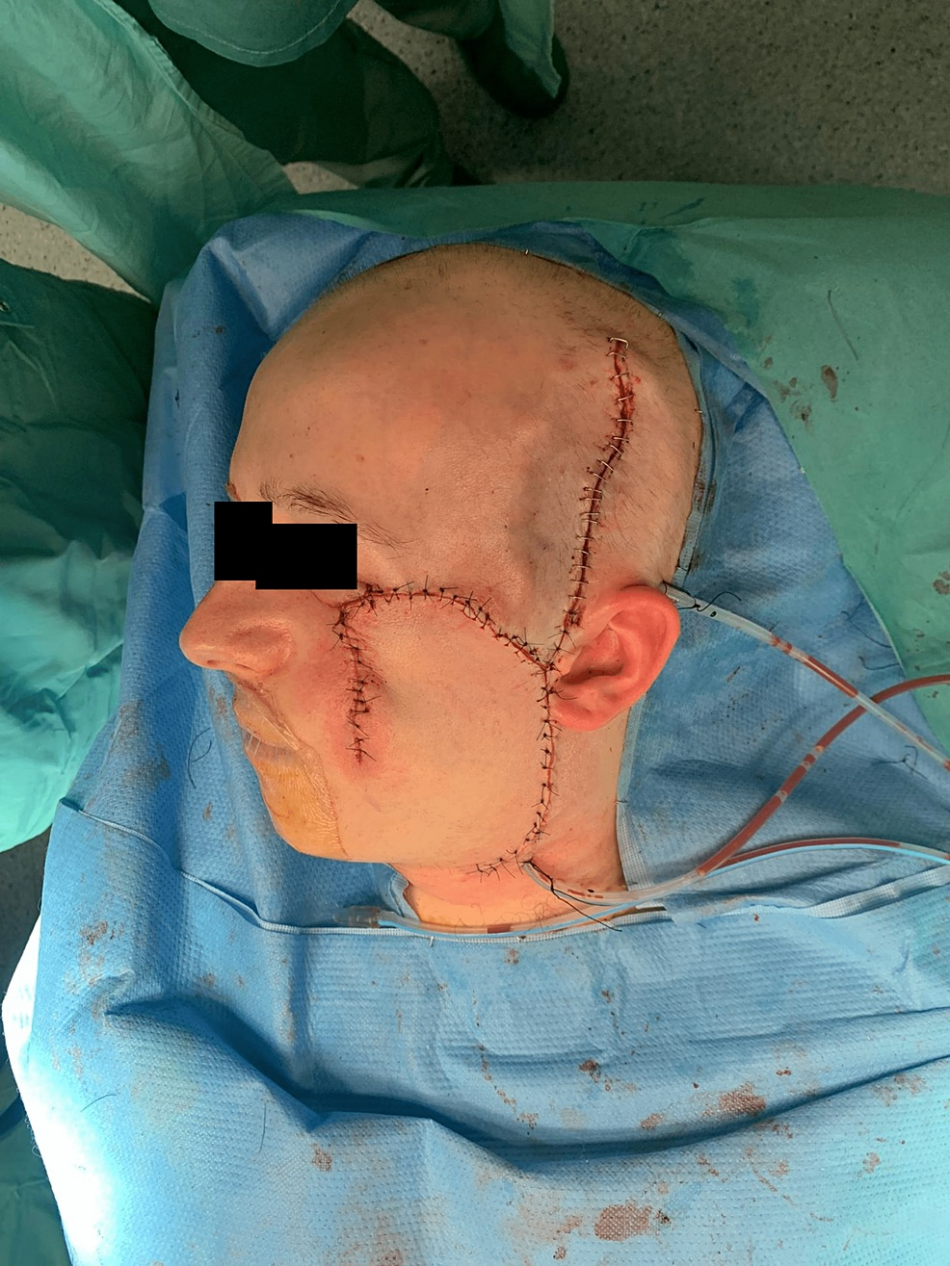
Mustardé flap

Further, a Mustardé flap was used to close the skin defect in the malar region (Figure 5). 

Pathological assessment of the surgical specimens revealed free surgical margins and a 60-70% response rate to neoadjuvant therapy. The patient was treated with ifosfamide (I) and etoposide (E) as adjuvants. Death occurred one year after surgery after showing clear signs of local recurrence.

## Discussion

James Ewing described Ewing´s sarcoma for the first time in 1921 [1,4]. It is uncommon for this type of tumor to affect the facial bones [3]. Zygoma involvement is even rarer with only a few cases reported in the English literature. Table 1 summarizes the reported cases.

**Table 1:** Summary of cases described in the literature Y, years; M, male; F, female; S, surgery; CT, chemotherapy; RT, radiotherapy

Author/References	Age/Gender	Location	Evolution	Symptoms	Treatment	Follow-up
Infante-Cossio et al. [1]	17Y/M	Zygoma	1 month	Swelling, No pain	CT+S	5 years
Deshingkar et al. [2]	15Y/F	Zygoma	1 month	Swelling, No pain	S	6 months
Rattana et al. [3]	19Y/M	Zygoma	1 month	Pain, Swelling	CT+S	Patient died 9 months after the diagnosis
El-Khayat et al. [4]	31Y/M	Zygomatic arch	2 months	Pain, Swelling	CT+S	2 years
Shibota et al. [5]	23Y/F	Zygomatic arch	-	Pain, Swelling	S+RT+CT	-
Posnick et al. [6]	Not available	Not available	Not available	Not available	Not available	Not available
Narasimhan et al. [7]	15Y/M	Zygoma	4 months	Swelling, No pain	CT + RT	10 months
Postovsky et al. [8]	2Y/F	Zygoma	1 month	Swelling, No pain	S + CT	9 months
Soni et al. [9]	17Y/F	Zygoma	3 months	Swelling, No pain	S+CT	7 months
Current case	21Y/M	Zygoma	7 months	Pain, Swelling	CT+S+CT	1 year

Ewing´s sarcoma is more prevalent among young people. Most cases occur between the ages of five and 25 years [9]. Patients' ages ranged from two to 31 years in our review. Both sexes are affected in the same proportion, but males have a slight prevalence in the head and neck regions [2,5]. This literature review describes the cases of four female and five male patients.

As the primary manifestation of Ewing sarcoma in the facial bones is rare, it is difficult to diagnose [1,2]. However, it is important to include this condition in the differential diagnosis for facial neoplasms with bone destruction [1]. The most common manifestation of the disease is the appearance of a mass [1,3]. The associated signs and symptoms are usually pain and inflammation with potential orbital involvement [1]. The evolution time until diagnosis ranges from one to seven months. Computed tomography is the best imaging modality [1,2]. Histological studies are insufficient to confirm this diagnosis; however, immunohistochemical staining, especially with CD-99, can help to improve the diagnosis [1,4].

The treatment requires a combination of systemic therapy and local control [10]. After a biopsy confirms the diagnosis, all patients at all clinical stages, should begin primary treatment, which may include up to nine cycles of induction chemotherapy, followed by local control therapy and adjuvant treatment. The average duration of treatment is 10-12 months. [11,12]. Systemic chemotherapy is a multiagent treatment. The VDC/IE regimen, including vincristine (V), doxorubicin (D), cyclophosphamide (C), ifosfamide (I), and etoposide (E), is the preferred first-line treatment [10-12]. Local control therapy after primary treatment should be chosen individually based on the tumor location, size, response to chemotherapy, patient age, and anticipated morbidity. Two major options are wide excision and definitive radiotherapy with chemotherapy [11]. The goal of local therapy is to treat the entire volume of tissue involved. Surgery is the best treatment for the resection of lesions with safe margins, and all tissues originally involved must be excised [1,12]. Moreover, a study showed that the risk of local recurrence was higher when radiotherapy was used alone [12]. The role of radiotherapy in Ewing´s sarcoma of the facial bones remains debatable because it may cause functional sequelae and local complications, depending on its location [1,2,4]. Radiotherapy with definitive intent should be used only if complete surgical excision is not possible or in cases where surgery is unacceptably morbid [12]. This sequence of treatment is well-defined for Ewing's sarcoma in other locations, such as long bones. However, the treatment sequence for Ewing´s sarcoma of the facial bones described in the literature varies [1]. 

In our review, two patients underwent surgery followed by adjuvant chemotherapy, three underwent neoadjuvant chemotherapy followed by surgery, one underwent surgery followed by radiotherapy and chemotherapy, one underwent chemotherapy and radiotherapy, and one underwent surgery only [1-9]. We report a case in which the patient received neoadjuvant chemotherapy, and the tumor progressed during treatment, which could suggest some chemotherapy resistance. According to these guidelines, the best time to begin local control treatment should be discussed at a multidisciplinary level, considering the primary site, size, response, expected morbidity from surgery, and tolerability [12]. Therefore, in this case, because of the tumor´s location, rapid progression, and patient´s age, a multidisciplinary team decided to perform radical surgery. The patient had completed nine weeks of neoadjuvant chemotherapy without response; therefore, extending or changing the neoadjuvant treatment regimen was not indicated, and there was a risk of losing the indication for surgery. Advances in craniofacial and reconstructive surgery have allowed for combined resection and reconstruction [1]. In the present case, the tumor primarily affected the zygomatic body. PEEK prostheses are excellent options for reconstructive surgery.

Some authors have argued that maxillofacial lesions have a better prognosis than lesions in other locations. This is because local signs, such as swelling, are more visible [2,3,9]. Although the treatment is well-defined in other locations, the best treatment sequence for maxillofacial tumors remains controversial. Recurrence occurs in approximately 30- 40% of patients and has a very poor prognosis. Patients with a longer time between recurrences have a better chance of survival after recurrence [11]. Other known risk factors for poor prognosis include tumor volume, lactate dehydrogenase (LDH) levels, axial localization, older age (>15 years), a poor histological response to preoperative chemotherapy, and incomplete or no surgery at the primary site. Recurrent Ewing´s sarcoma, is almost always fatal, although additional responses to chemotherapy are common and potentially beneficial [12].

## Conclusions

We reported a case of Ewing´s sarcoma of the zygoma in a young male, which is infrequently reported in the literature. Multidisciplinary treatment and strict follow-up are crucial for disease control in this type of tumor. Preoperative neoadjuvant chemotherapy combined with a radical surgical resection, followed by adjuvant chemotherapy, is a good treatment option. However, in our case, the tumor size increased during neoadjuvant treatment, which could have made the tumor unresectable or required a more deforming resection.
